# Nature-based early childhood education for child health, wellbeing and development: a mixed-methods systematic review protocol

**DOI:** 10.1186/s13643-020-01489-1

**Published:** 2020-10-02

**Authors:** Avril Johnstone, Paul McCrorie, Rita Cordovil, Ingunn Fjørtoft, Susanna Iivonen, Boris Jidovtseff, Frederico Lopes, John J. Reilly, Hilary Thomson, Valerie Wells, Anne Martin

**Affiliations:** 1 MRC/CSO Social and Public Health Sciences Unit, University of Glasgow, Berkeley Square, 99 Berkeley Street, Glasgow, G3 7HR, Scotland; 2 CIPER, Faculdade de Motricidade Humana, Universidade de Lisboa, Estrada da Costa, 1499-002 Cruz Quebrada, Lisbon, Portugal; 3 Faculty of Humanities, Sports and Education Sciences, University of South-Eastern Norway, Notodden, Norway; 4 School of Applied Educational Science and Teacher Education, University of Eastern Finland, Joensuu, Finland; 5 Research Unit on Childhood, Department of Sport and Rehabilitation Sciences, University of Liege, 2 Allee des sports, 4000 Liege, Belgium; 6 Laboratory of Motor Behavior, Faculdade de Motricidade Humana, Universidade de Lisboa, Lisbon, Portugal; 7 School of Psychological Sciences and Health, University of Strathclyde, 50 George Street, Glasgow, G1 1QE Scotland

**Keywords:** Early childhood education, Children, Health, Wellbeing, Development

## Abstract

**Background:**

Several systematic reviews have reviewed the evidence relating to nature on aspects of children and adolescent’s health and wellbeing; however, none have looked at the associations or effectiveness of attending nature-based early childhood education (ECE). The main objective is to systematically review and synthesise the evidence to determine if nature-based ECE enhances children’s health, wellbeing and development.

**Methods:**

We will search the following electronic databases (from inception onwards): MEDLINE, Scopus, PsycINFO, ERIC, SportDiscus, Australian Education Index, British Education Index, Child Development and Adolescent studies, and Applied Social Sciences Index and Abstracts. Grey literature will be identified searching dissertations and reports (e.g. Open Grey, Dissertations Theses Database [ProQuest], and Google Scholar). All types of studies (quantitative and qualitative) conducted in children (aged 2–7 years old) attending ECE who had not started education at primary or elementary school will be included. The exposure of interest will be nature-based ECE settings that integrate nature into their philosophy and/or curriculum and environment. The outcomes of interest will be all aspects of the child’s physical, cognitive, social and emotional health wellbeing and development. Two reviewers will independently screen full-text articles. The study methodological quality (or bias) will be appraised using appropriate tools. If feasible, a meta-analysis will be conducted using a random-effect model for studies similar in exposure and outcome. Where studies cannot be included in a meta-analysis, findings will be summarised based on the effect directions and a thematic analysis will be conducted for qualitative studies.

**Discussion:**

This systematic review will capture the state of the current literature on nature-based ECE for child health, wellbeing and development. The results of this study will be of interest to multiple audiences (including researchers and policy makers). Results will be published in a peer-reviewed journal. Gaps for future research will be identified and discussed.

**Systematic review registration:**

PROSPERO CRD42019152582

## Background

Time spent outdoors engaging in physical activity through active and outdoor play may be associated with higher levels of physical activity [1, 2] which is important for improving health outcomes, including fitness, weight management, bone density and mental wellbeing [3, 4]. However, children are increasingly engaging in low levels of physical activity and high amounts of sedentary time [5–7]. Given that physical activity levels decline around the time children start primary school [6, 8], it is important to intervene early and early childhood education (ECE) settings offer a potentially cost-effective and sustainable solution to addressing low levels of physical activity, promoting active and outdoor play and improving health outcomes [1, 3, 4, 7]. When children are outdoors in ECE settings, they engage in higher levels of physical activity compared to indoor settings [1, 7]. Furthermore, it is suggested that providing exposure to nature might provide additional benefits, including improved resiliency, mental wellbeing, motor development, prosocial behaviour and connection to nature [9–12].

Nature-based ECE is an umbrella term that encompasses different types of education, including nature-based preschool or kindergarten and forest kindergartens [13]. These types of nature-based ECE settings vary in approach, level of exposure and duration with some children spending most of the day in nature to once per week [13]. Nature is the common thread that ties these types of ECE settings together with features of nature integrated into their philosophy and design [13]. For example, trees, vegetation, natural loose-parts, rivers or ponds and other natural materials which children attending the ECE settings have access to.

The effects of engaging with nature in childhood are potentially wide-ranging and may extend beyond the health benefits of participating in active outdoor play. Recent literature reviews have suggested that engagement in nature improves a range of physical, social, emotional and cognitive outcomes [9, 10]. Two separate systematic reviews looking at the effects of nature more broadly (i.e. may or may not include education settings) have suggested improvements in emotional wellbeing, overall mental health, resilience, self-esteem and reduced stress in children and adolescents aged 0–18 years [14, 15]. A smaller number of studies also suggest improvements in learning, cognitive and social outcomes, such as cooperation and prosocial behaviour [14]. More evidence supports the effect of nature on children and adolescent’s physical activity, particularly moderate-to-vigorous intensity physical activity (MVPA) [14]. However, included studies generally have unclear or a high risk of bias (particularly incomplete outcome data and selective reporting), and across most outcomes meaning that we need to infer findings with caution. Furthermore, evidence thus far exists primarily in the early adolescent age groups than in the preschool age. Only 3% of the eligible 90 individual studies included in the Mygind et al. [14] systematic review included participants 3–7 years old, and only 3 of 35 studies in the Tillmann et al. [15] systematic review look at children < 7 years. In summary, systematic reviews published looking at nature on aspects of children’s health and wellbeing have focussed on both educational and non-educational settings (e.g. community) and predominately in the early adolescent age group. To our knowledge, no systematic review exists that looks solely at whether nature-based ECE improves young children’s (2–7 years) health, wellbeing and development. This systematic review will summarise key findings and enhance the quality of future research.

If nature-based ECE is to become more prevalent in the UK and globally, the evidence must be synthesised to identify the strengths and weaknesses that exist, and the gaps that must be addressed. Therefore, the aim of this research project is to systematically review and synthesise the published and unpublished evidence to:

- Determine if attending nature-based ECE is associated or has an effect on children’s health, wellbeing and development.
- Explore children’s, parent’s and/or practitioner’s perceptions of nature-based ECE on children’s health, wellbeing and development.

## Methods

The systematic review protocol was registered to the International Prospective Register of Systematic Reviews (CRD42019152582). This study protocol is being reported in accordance with the reporting guidance provided in the Preferred Reporting Items for Systematic Reviews and Meta-Analyses Protocols (PRISMA-P) statement (see checklist in Additional file 1) [16, 17]. The subsequent section aims to provide an overview of the methodology used. The proposed systematic review will be reported in accordance with the reporting guidance provided in the Preferred Reporting Items for Systematic Reviews and Meta-Analyses (PRISMA) guidelines will be followed [18].

### Information sources and search strategy

Nine relevant electronic databases will be searched (from inception onwards): (1) Education Research Information Centre (ERIC), (2) Australian Education Index, (3) British Education Index, (4) Child Development and Adolescent studies, (5) Applied Social Sciences Index and Abstracts, (6) PsycINFO, (7) MEDLINE, (8) SportDiscus and (9) Scopus.

Grey literature such as dissertations and reports will be searched in Open Grey (www.opengrey.eu), Dissertation and Theses Database (ProQuest) and Directory of Open Access Journals (www.doaj.org). Google Scholar will be searched, and the first 10 pages checked. Websites of relevant organisations, professional bodies and other groups involved in outdoor education, outdoor play and green space development will be searched. Relevant organisations, practitioners and researchers in the field will also be contacted to obtain information.

Search strategies will be constructed by the lead author (AJ) and an information scientist (VW, co-author) with support from co-authors who have expertise in fields related to nature, health, wellbeing, development, education and systematic review methodology. Relevant systematic reviews and publications will be reviewed for key words and related terms will be considered to develop a comprehensive search strategy. The strategy will be tested and refined until a finalised search strategy is developed. Once the search strategy has been finalised, it will be adapted for each database and other web searches and the literature search will not be restricted by year of publication or language. A draft search strategy for MEDLINE is provided in Additional file 2. References will be imported to Endnote and one reviewer (AJ) will remove duplicates.

### Eligibility criteria and selection procedure

Titles and abstracts will be screened once (AJ, PM, RC, IF, SI, FL, BJ, VW) and 10% of the titles and abstract will be screened in duplicate independently (AM). Two researchers will then independently screen full text articles in duplicate. In instances when reviewers may not agree during any part of the screening process, a third reviewer will be brought in to discuss and resolve the disagreement. Where there are multiple publications for the same study, we will combine and report all publications as a single study.

The selection criteria will follow the PI(E)COS (Population, Intervention or Exposure, Comparison, Outcomes and Study design) framework.

#### Population

Children attending ECE settings and who have not started education at primary or elementary school will be included in the systematic review. Age ranges for children attending ECE vary in each country, but children typically attend ECE settings between 2 and 7 years. Studies which include children < 2 years or > 7 years will be excluded because this age group would not typically attend ECE. In retrospective study designs, children can be > 7 years if the study focuses on the time the children attended ECE. We will use mean age, range or median reported in the study to decide whether the study is eligible. If a study is conducted in an ECE setting, but no age is reported, it will be included. Studies which include a child population from disease conditions (for example, autism, physical disability, attention deficit hyperactivity disorder) only will be excluded.

#### Exposure/intervention

The exposure of interest is nature-based ECE which is an umbrella term that encompasses all nature based ECE settings, including nature-based preschool, kindergarten, day care and nursery [13]. These types of nature-based ECE settings vary in approach, level of exposure and duration and could include full day nature-based ECE, interventions enhancing the amount and quality of natural elements (e.g. planting trees and vegetation) in the ECE setting or the association of natural elements (e.g. hills, trees, water, snow etc.). These will be identified in the literature if the ECE setting integrates nature into their philosophy and/or curriculum and environment or the authors describe their studies as using natural elements such as, trees, sand, water, snow, natural loose-parts and hills. ECE settings where nature is not the predominant exposure, i.e. they do not integrate nature into their philosophy and/or curriculum and environment, or the authors do not describe their studies as using natural elements will be excluded. For example, studies where the ECE setting utilises a more traditional indoor approach or where the playground is predominately concrete and features manmade structures (swings, slide, climbing frame etc.) will be excluded.

#### Comparison

Attendance of traditional ECE, such as preschool and childcare. These education settings tend to provide outdoor opportunities for play, but they are not nature-based in philosophy or design. Children who attend traditional ECE settings might spend less time outdoors and the outdoor environment tends to be manufactured with elements such as swings, slide and climbing frames.

#### Outcomes

Any child-level outcome related to health, wellbeing and development. These are broad terms but would include outcomes related to all aspects of the child’s physical (e.g. physical activity, motor skills), cognitive (e.g. executive functions, attention), social (e.g. pro-social behaviour, connectedness to nature) and emotional (e.g. stress reduction) health, wellbeing and development. Studies will be excluded if they include outcomes which are not child-level (for example, impact on practitioners or changes to the ECE setting) and studies using unvalidated questionnaires will be excluded (for both quantitative and qualitative designs).

#### Study designs

Quantitative and qualitative primary research designs will be considered. Qualitative studies which explore perceptions (from parent, practitioner or child) at a time when the child was attending the nature-based ECE setting will be included. All quantitative study designs will be considered, including cross-sectional and case-control studies measured when the child was attending nature-based ECE; longitudinal, quasi-experimental and experimental studies with at least two time points; and retrospective studies if outcomes were assessed at a time when the child attended the nature-based ECE setting. Studies will be excluded where the time point of outcome measurement cannot be readily associated with the exposure, for example, if studies measure effect once the child has left the nature-based ECE or case studies reviewing only one child.

### Data extraction

#### Quantitative data

Data will be extracted from included studies using a pre-defined (see Additional file 3) and piloted data extraction template by one reviewer with another reviewer cross-checking all extracted data. In instances where data might be missing, or additional information required for the eligible studies, the study authors will be contacted to provide the relevant information. An email will be sent to the corresponding and lead author requesting the required information. If they do not respond initially, a reminder email will be sent 2 weeks after the initial email. If they have not responded within 1 month from initial contact their study may be excluded from the systematic review or possible meta-analysis.

The following data will be extracted:

- Study ID (authors, year of publication)
- Country
- Study design (randomised controlled trial [RCT], cross-sectional etc.)
- Participants (age, gender, socio-economic status, sample size etc.)
- Intervention/exposure type and duration (forest school, playground modifications etc.). Details on what any possible comparator groups received will also be detailed (for example, characteristics of traditional preschool).
- Outcome measures (type, assessment tool, unit and time point of assessment etc.)
- Outcomes and results (effect estimates, standard deviation, confidence intervals, effect direction etc.)

#### Qualitative data

One reviewer will read through each eligible qualitative study and provide a summary of the main themes as reported by the study author and any other relevant information. A second reviewer will read the study and summary provided by reviewer one and add any additional information.

The following data will be extracted:

- Study ID (authors, year of publication)
- Country
- Participants (i.e. gender, socio-economic status, sample size)
- Intervention/exposure type
- Intervention/exposure duration
- Research aims
- Outcome measures (interviews, focus groups etc.)
- Outcomes and results (summary of key themes).

Inclusion of qualitative data aims to complement quantitative findings by explaining potential confounding factors and pathways of nature-based ECE on children’s health wellbeing and/or development, or to evidence outcomes that may not be reported in quantitative studies. Synthesis of qualitative data is not intended to be detailed, but instead to identify and map the main themes in each study.

### Quality appraisal of included studies

The quality of all included studies will be assessed by at least two reviewers independently, cross-checked and disagreement resolved through discussion with a third reviewer. The quality of quantitative studies will be assessed using the well-established Effective Public Health Practice Project (EPHPP) Quality Assessment Tool [19]. Small modifications will be made to this tool to ensure it is relevant to the present review, for example, defining target population, specifying confounders of interest and enhancing the overall rating of the paper. For qualitative data, the trustworthiness of the study will be assessed using the Dixon-Woods checklist [20]. Qualitative studies will be excluded if the research questions are not suited to qualitative inquiry (question 2) or if the paper does not make a useful contribution to the review question (question 7). The justification for excluding studies based on these questions is because findings cannot be trusted, for example, if the study has not been described with sufficient detail to ensure trustworthiness in the methodology used.

A draft of the quality assessment tools can be found in Additional file 4.

### Data synthesis

Continuous and dichotomous data will be treated separately. Where possible, we will convert the effect data to odds ratios for dichotomous outcomes and standardised mean difference for continuous outcomes. We will consider using a meta-analysis to calculate an overall effect size estimate where more than one study of a similar design reports data on the same outcome domain (e.g. MVPA), studies have a reasonable sample size and statistical heterogeneity (*i*^2^) is < 50%. The meta-analysis will use a random-effect model (as study characteristics and/or treatment effects are expected to be heterogenous) and will be conducted using appropriate software (e.g. Comprehensive Meta-Analysis Software © or Review Manager ©). To test the robustness of our findings and conclusions, we will conduct sensitivity analyses where we remove studies of high risk of bias (i.e. poor study quality) from the analysis. If the type and amount of data allow, we will conduct subgroup analyses to investigate differential associations and/or effects of the following: differences by age (2–5 years; 5–7 years), differences between girls and boys, different time points of outcome assessment, different durations of time spent in nature-based ECE (half day vs full day; number of days) and level of exposure to nature (high exposure to nature vs minimal exposure to nature). For exposure to nature, we recognise there is ambiguity in the literature on optimum exposure levels and often the description of the exposure is limited in the studies. Therefore, eligible studies will be reviewed on the description of the exposure and divided into studies that are clearly described as having a high amount of exposure (i.e. nature-based ECE setting entirely in a wooded area) to those where description is limited or the exposure is minimal (i.e. a few trees, small amount of grass area).

Where the use of meta-analysis of standardised effect sizes results in exclusion of most included studies for any single outcome domain, we will perform a Synthesis Without Meta-analysis (SWiM) based on effect direction [21]. For effect direction, a summary table will be presented where studies will be ordered by quality to prioritise the best evidence. Outcomes will be grouped by similar outcome domains: physical (physical activity, motor development, sleep), cognitive (executive functions, attention, creativity), social (pro-social behaviour connectedness to nature) and emotional (stress reduction). Where the synthesis is based on effect direction, the synthesis will address a question of whether this is evidence of a positive or negative effect. Confidence intervals and effect sizes will be prioritised to interpret findings and when these are not presented, *p* values be used. In addition to an effect direction plot, a narrative synthesis will also be conducted to report on findings grouped by outcome domains, as described above. Any conclusions drawn will be based on better quality evidence.

For qualitative studies, a thematic analysis of reported themes will be conducted, grouping them into lower and higher order themes. A summary of the findings of the qualitative and quantitative studies will be combined into single logic model which will be developed by two reviewers. The purpose of the logic model is to present a testable theory of change that will allow comparison and examination of how the different data types relate to each other and to enable readers to identify gaps for future research.

#### Certainty of quantitative evidence

We will use The Grading of Recommendations Assessment, Development and Evaluation (GRADE) framework to assess the certainty of the evidence across studies at an outcome level [22]. Where there are two or more studies reporting on the same outcome, we will assess the risk of bias, precision, consistency and directness. The certainty of evidence will be rated up or down depending on these criteria to provide an overall rating for the certainty of the evidence: very low (true effect different from estimated effect, very likely to change with new evidence emerging), low, moderate and high (true effect is similar to estimated effect; unlikely to change with new evidence emerging) [22].

## Discussion

This protocol presents the planned methodology for a systematic review covering both published and unpublished quantitative and qualitative studies that aim to determine the effects of nature based ECE on all (or different) aspects of children’s health, wellbeing and development. A narrative synthesis approach will be conducted to report the findings and where possible, a meta-analysis will be conducted to provide a more robust evidence base for nature-based ECE. Limitations of the studies included in this review will be reported which we envisage will be related to the study designs (predominately cross-sectional), small sample sizes and selection bias. The systematic review is also likely to have a number of limitations, such as data extraction will not be performed in duplicate, the quality assessment tools may be limited and meta-analyses are likely to be conducted in a small number of studies only. If any changes are made to this protocol, these will be described in the published systematic review.

This systematic review will present the effect of nature-based ECE on a range of children’s health, wellbeing and development outcomes. We hope that these findings will present gaps for future areas of research, inform researchers and policy makers and impact on ECE practice. Dissemination of the findings from this systematic review will involve a published manuscript, a report for policy makers and ECE practitioners, conferences and other relevant presentations.

## Supplementary information

**Additional file 1.** PRISMA P checklist.

**Additional file 2.** Example search strategy for Medline.

**Additional file 3.** Data extraction template – Quantitative and Qualitative

**Additional file 4.** Modified Effective Public Health Practice Project 444 (EPHPP) Quality Assessment Tool and Dixon-Woods (2004) Checklist.

## Data Availability

Not applicable.

## Abbreviations

ECE: Early childhood education
MVPA: Moderate-to-vigorous intensity physical activity
PRISMA: Preferred Reporting Items for Systematic Reviews and Meta-Analyses
ERIC: Education Research Information Centre
PI(E)COS: Population, Intervention or Exposure, Comparison, Outcomes and Study design)
RCT: Randomised controlled trial
EPHPP: Effective Public Health Practice Project
SWiM: Synthesis Without Meta-analysis
UV: Ultraviolet
GRADE: Grading of Recommendations Assessment, Development and Evaluation
