# Supplementary material for: Nature-based early childhood education for child health, wellbeing and development: a mixed-methods systematic review protocol
Source: Syst Rev. 2020 Oct 2;9:226. doi: 10.1186/s13643-020-01489-1 (PMC7532588; doi:10.1186/s13643-020-01489-1)
Supplement: Supplementary file 2 — Additional file 2. Example search strategy for Medline. [file 13643_2020_1489_MOESM2_ESM.docx]

**Additional file 2.**

**Example search strategy for the Medline database.**

| S1 | (MH "Child, Preschool") |
| --- | --- |
| S2 | TI child* OR AB child* |
| S3 | TI (boy* OR girl*) or AB (boy* OR girl*) |
| S4 | TI toddler OR AB toddler |
| S5 | TI young N1 child* OR AB young N1 child* |
| S6 | TI early N1 child* OR AB early N1 child* |
| S7 | TI early N1 year* OR AB early N1 year* |
| S8 | TI “pre-primary” or AB “pre-primary” |
| S9 | S1 OR S2 OR S3 OR S4 OR S5 OR S6 OR S7 OR S8 |
| S10 | (MH "Schools, Nursery") |
| S11 | TI nurser* OR AB nurser* |
| S12 | (MH "Learning") OR TI early N1 learning OR AB early N1 learning |
| S13 | TI (“preschool” or “pre-school”) OR AB (“preschool” or “pre-school”) |
| S14 | TI kindergarten OR AB kindergarten |
| S15 | TI (childcare OR child N1 care) OR AB (childcare OR child N1 care) |
| S16 | TI (daycare OR day N1 care) OR AB (daycare OR day N1 care) |
| S17 | (MH "Education") OR TI (education OR "preschool education" OR "outdoor education" OR "adventure education") OR AB (education OR "preschool education" OR "outdoor education" OR "adventure education") |
| S18 | MM "Play and Playthings" OR TI (Play OR “play-based learning”) OR AB (Play OR “play-based learning”) |
| S19 | TX (Waldkindergartens OR udeskole OR friluftsliv OR peuterspeelzaal OR kinderopvang OR bush N1 kinder*) OR TI (forest N1 kindergarten* OR forest N1 school*) OR AB (forest N1 kindergarten* OR forest N1 school*) |
| S20 | S10 OR S11 OR S12 OR S13 OR S14 OR S15 OR S16 OR S17 OR S18 OR S19 |
| S21 | TI outdoor* OR AB outdoor* |
| S22 | TI (nature OR “nature-based”) OR AB (“nature-based”) |
| S23 | TI environment* OR TI outdoor N1 environment* OR AB outdoor N1 environment* |
| S24 | TI (forest* OR wood* OR park* OR recreation* OR landscape* OR tree* OR hill* OR garden* OR beach* OR eco) |
| S25 | AB (forest* OR wood* OR park* OR recreation* OR landscape* OR tree* OR hill* OR garden* OR beach* OR eco) |
| S26 | TI (green OR greenspace or green N1 space) OR AB (green OR greenspace or green N1 space) |
| S27 | TI (loose N1 parts OR “loose-parts”) OR AB (loose N1 parts OR “loose-parts”) |
| S28 | TI (adventure* OR wild OR “open-air”) OR AB (adventure* OR wild OR “open-air”) |
| S29 | S21 OR S22 OR S23 OR S24 OR S25 OR S26 OR S27 OR S28 |
| S30 | S9 AND S21 AND S30 |
