# Supplementary material for: Nature-based early childhood education for child health, wellbeing and development: a mixed-methods systematic review protocol
Source: Syst Rev. 2020 Oct 2;9:226. doi: 10.1186/s13643-020-01489-1 (PMC7532588; doi:10.1186/s13643-020-01489-1)
Supplement: Supplementary file 3 — Additional file 3. Data extraction template – Quantitative and Qualitative [file 13643_2020_1489_MOESM3_ESM.docx]

**Additional file 3.**

**Data extraction template - Quanitative**

| **STUDY DETAILS** | **POPULATION** | | **INTERVENTION / EXPOSURE** | | | | | **OUTCOMES** | | | **FINDINGS** | | | |
| --- | --- | --- | --- | --- | --- | --- | --- | --- | --- | --- | --- | --- | --- | --- |
| Study ID (author, year, country) & study design | Age (range &/or mean±SD), sex (n or % m/f), SES | Sample size (n) – intervention & control | Exposure Type | Exposure Description (Intervention & Control) | Exposure Measurement Type & description (questionnaire details etc.) | Units | Duration of intervention & follow-up | Outcome Description & measurement | Outcome Units | Outcome Validity/ Reliability ? | Findings (Means - Pre-to-Post Intervention & control) (mean difference, 95% CI or SE or SD) | Associations/ relationships (if applicable) | Findings (Between-Group Comparison) (mean difference, 95% CI or SE or SD) | Covariates & details of analysis |
|  |  |  |  |  |  |  |  |  |  |  |  |  |  |  |

**Data extraction template – Qualitative**

| **STUDY DETAILS** | **POPULATION** | | | **INTERVENTION / EXPOSURE** | | | **OUTCOMES AND METHOD** | | | | **FINDINGS** | |
| --- | --- | --- | --- | --- | --- | --- | --- | --- | --- | --- | --- | --- |
| Study ID (author, year, country) & study design | Age (range & mean±SD), sex (n or % m/f), SES | Sample size (n) – intervention and control | Sample Selection | Exposure Type | How is Exposure Type defined by authors | Level of exposure | Research aims | Data collection method (focus groups etc.) | Details of analysis | Year interviews were conducted | Summary of Findings | Key themes reported |
|  |  |  |  |  |  |  |  |  |  |  |  |  |
