# Supplementary material for: Nature-based early childhood education for child health, wellbeing and development: a mixed-methods systematic review protocol
Source: Syst Rev. 2020 Oct 2;9:226. doi: 10.1186/s13643-020-01489-1 (PMC7532588; doi:10.1186/s13643-020-01489-1)
Supplement: Supplementary file 4 — Additional file 4. Modified Effective Public Health Practice Project 444 (EPHPP) Quality Assessment Tool and Dixon-Woods (2004) Checklist. [file 13643_2020_1489_MOESM4_ESM.docx]

**Additional file 4.**

**Modified Effective Public Health Practice Project (EPHPP) Quality Assessment Tool**

A) SELECTION BIAS

(Q1) Are the individuals selected to participate in the study likely to be representative of the target population? (i.e. children aged 2-7 years not in formal education yet)

1. Very likely
2. Somewhat likely
3. Not likely
4. Can’t tell

(Q2) What percentage of selected individuals consented to the research?

1. 80 - 100% agreement
2. 60 – 79% agreement
3. less than 60% agreement
4. Not applicable
5. Can’t tell

| **RATE THIS SECTION** | **STRONG** | **MODERATE** | **WEAK** |
| --- | --- | --- | --- |
| See dictionary | 1 | 2 | 3 |

B) STUDY DESIGN

Indicate the study design:

1. Randomized controlled trial
2. Controlled clinical trial
3. Cohort analytic (two group pre + post)
4. Case-control
5. Cohort (one group pre + post (before and after))
6. Interrupted time series
7. Other specify ____________________________
8. Can’t tell

Was the study described as randomized? If NO, go to Component C.

**No Yes**

If Yes, was the method of randomization described? (See dictionary)

**No Yes**

If Yes, was the method appropriate? (See dictionary)

**No Yes**

| **RATE THIS SECTION** | **STRONG** | **MODERATE** | **WEAK** |
| --- | --- | --- | --- |
| See dictionary | 1 | 2 | 3 |

C) CONFOUNDERS

(Q1) Were there important differences between groups prior to the intervention?

1. Yes
2. No
3. Can’t tell

The following are examples of confounders:

1. Gender
2. Age
3. Socio economic status (SES – e.g. Parental education, deprivation status)

(Q2) If yes, indicate the percentage of relevant confounders that were controlled (either in the design (e.g. stratification, matching) or analysis)?

1. All confounders
2. Two confounders
3. One confounder
4. Can’t Tell

| **RATE THIS SECTION** | **STRONG** | **MODERATE** | **WEAK** |
| --- | --- | --- | --- |
| See dictionary | 1 | 2 | 3 |

D) BLINDING

(Q1) Was (were) the outcome assessor(s) and/or analysists aware of the intervention or exposure status of participants?

1. Yes
2. No
3. Can’t tell

(Q2) Were outcome assessors aware of the research question?

1. Yes
2. No
3. Can’t tell

| **RATE THIS SECTION** | **STRONG** | **MODERATE** | **WEAK** |
| --- | --- | --- | --- |
| See dictionary | 1 | 2 | 3 |

E) DATA COLLECTION METHODS

(Q1) Were data collection tools shown to be valid?

1. Yes
2. No
3. Can’t tell

(Q2) Were data collection tools shown to be reliable?

1. Yes
2. No
3. Can’t tell

| **RATE THIS SECTION** | **STRONG** | **MODERATE** | **WEAK** |
| --- | --- | --- | --- |
| See dictionary | 1 | 2 | 3 |

F) WITHDRAWALS AND DROP-OUTS

(Q1) Were withdrawals and drop-outs reported in terms of numbers and/or reasons per group?

1. Yes
2. No
3. Can’t tell
4. Not Applicable (i.e. one time surveys or interviews)

(Q2) Indicate the percentage of participants completing the study. (If the percentage differs by groups, record the lowest).

1. 80 -100%
2. 60 - 79%
3. less than 60%
4. Can’t tell
5. Not Applicable (i.e. Retrospective case-control)

| **RATE THIS SECTION** | **STRONG** | **MODERATE** | **WEAK** |
| --- | --- | --- | --- |
| See dictionary | 1 | 2 | 3 |

**COMPONENT RATINGS**

**Please transcribe the information from the grey boxes on pages 1-3 onto this page. See dictionary on how to rate this section.**

| **A** | **SELECTION BIAS** | **STRONG** | **MODERATE** | **WEAK** |
| --- | --- | --- | --- | --- |
|  |  | 1 | 2 | 3 |
| **B** | **STUDY DESIGN** | **STRONG** | **MODERATE** | **WEAK** |
|  |  | 1 | 2 | 3 |
| **C** | **CONFOUNDERS** | **STRONG** | **MODERATE** | **WEAK** |
|  |  | 1 | 2 | 3 |
| **D** | **BLINDING** | **STRONG** | **MODERATE** | **WEAK** |
|  |  | 1 | 2 | 3 |
| **E** | **DATA COLLECTION METHOD** | **STRONG** | **MODERATE** | **WEAK** |
|  |  | 1 | 2 | 3 |
| **F** | **WITHDRAWALS AND DROPOUTS** | **STRONG** | **MODERATE** | **WEAK** |
|  |  | 1 | 2 | 3 |

**Overall Grade (based on above six criteria):**

| - Scored **1** for study design (i.e. controlled studies); AND - Scored **1** or **2** in at least three other important components, including:   - selection bias   - confounders   - blinding   - withdrawals and drop-outs. | **STRONG**  **1** |
| --- | --- |
| - Scored **1** for study design; AND - Scored **1** or **2** in two other important components, including:   - selection bias   - confounders   - blinding   - withdrawals and drop-outs.   OR   - Scored **2** for study design; AND - Scored **1** or **2** in at least three other important components, including:   - selection bias   - confounders   - blinding   - withdrawals and drop-outs. | **MODERATE**  **2** |
| - Scored **1** for study design; AND - Scored **3** in more than two other important components, including:   - selection bias   - confounders   - blinding   - withdrawals and drop-outs.   OR   - Scored **2** for study design; AND - Scored **3** in more than one other important components, including:   - selection bias   - confounders   - blinding   - withdrawals and drop-outs.   OR   - Scored **3** for study design | **WEAK**  **3** |

**Dixon-Woods (2004) checklist**

| **Question 1** | Are the research questions clear? |
| --- | --- |
| **Question 2** | Are the research questions suited to qualitative inquiry |
| **Question 3** | Are the following clearly described?   - Sampling - Data collection - Analysis |
| **Question 4** | Are the following appropriate to the research question?   - Sampling - Data collection - Analysis |
| **Question 5** | Are the claims made supported by sufficient evidence? |
| **Question 6** | Are the data, interpretations, and conclusions clearly integrated? |
| **Question 7** | Does the paper make a useful contribution to the review question? |
